# Peripheral Artery Disease Causes More Harm to Patients than COVID-19

**DOI:** 10.3390/healthcare10101809

**Published:** 2022-09-20

**Authors:** Mohammad Mahdi Kasiri, Martina Mittlboek, Giurgiana-Aura Giurgea, Norbert Fortner, Philip Lirk, Wolf Eilenberg, Bernd Gollackner, Christoph Neumayer

**Affiliations:** 1 Department of General Surgery, Division of Vascular Surgery, Medical University of Vienna, 1090 Vienna, Austria; 2 Section for Clinical Biometrics, Centre for Medical Statistics, Informatics, and Intelligent Systems, Medical University of Vienna, 1090 Vienna, Austria; 3 Department of Internal Medicine II, Clinical Divisions of Angiology, Medical University of Vienna, 1090 Vienna, Austria; 4 Department of Anesthesiology, Perioperative and Pain Medicine, Brigham and Women’s Hospital, Harvard Medical School, Boston, MA 02115, USA

**Keywords:** COVID-19, SARS-CoV-2, pandemic, peripheral artery disease, vascular disorders, amputation

## Abstract

Background: To optimize our strategic planning, we aimed to investigate the impact of the COVID-19 pandemic on the treatment of patients with peripheral artery disease (PAD) at our tertiary care hospital. Methods: We performed a retrospective single-center cohort study. In total, 1210 patients were included: 611 patients admitted between March and December 2020, compared to retrospective data from 599 patients from the same period in 2019. Results: Emergency admissions involving patients with advanced stage PAD increased significantly during the pandemic period of 2020, compared to the same period in 2019 (*p* < 0.0098). This increase was accompanied by increased limb amputations performed during the first lockdown, post-lockdown and the second lockdown in 2020, compared to respective time periods in 2019 (*p* < 0.0003, *p* < 0.0004, *p* = 1). No SARS-CoV-2 infection was observed among patients with PAD during the observation period. Conclusions: Strict lockdown protocols adversely affected the care of PAD patients, with persisting aftereffects, including increased emergency admission with unsuccessful revascularization attempts leading to limb amputation, even after the peak of the pandemic had passed. We believe that providing continuous care to PAD patients, even in times of global pandemics, will prevent the unfavorable outcomes observed during the COVID-19 pandemic in 2020.

## 1. Introduction

Peripheral artery disease (PAD), one of the most prevalent manifestations of atherosclerotic vascular disorder, affects more than 200 million individuals with a rapid increasing frequency worldwide [1]. It encompasses pathological processes that alter the normal structure and function of peripheral arteries, leading to stenosis, occlusion or/and aneurysmatic alteration [2]. Moreover, it impairs patient’s quality of life and has been proposed as a major burden of vascular patients (VPs), due to associated ischemic events, which may require subsequent limb amputation [3]. In addition, PAD shows a higher prevalence and mortality rate when compared with pandemic viral infections, such as HIV [4]. Therefore, great focus is required on the management of this disease entity, especially in times of pandemic.

However, to our knowledge, scarce evidence exists on the prevalence of severe acute respiratory syndrome coronavirus 2 (SARS-CoV-2) infection in patients with PAD. Nevertheless, due to the presence of predisposing factors for developing severe SARS-CoV-2 infection, including cardiac disorders, diabetes, and hypertension, the follow-up care for patients with PAD was discontinued during the COVID-19 pandemic in 2020. Instead, all resources were dedicated to protecting patients from the pandemic, and the rising burden of the underlying PAD was neglected. In the first lockdown (LD) period of the COVID-19 pandemic, concerns about the shortage of health care resources necessary to manage the pandemic abounded, which led to consequential changes in daily clinical practice. These included split-team policy, postponement of outpatient appointments, scaling down and rescheduling elective surgeries to after the LD period, and conducting only emergency surgeries. At first glance, these protocols led to a temporary decrease in the daily SARS-CoV-2 infection rate among the general population, but ended up causing harm to patients with advanced PAD, regardless of their SARS-CoV-2 infection status [5,6].

Despite growing knowledge about the management of cardiac disorders, limited data exist concerning the care of patients with PAD during the COVID-19 pandemic. Therefore, we sought to investigate the impact of the COVID-19 pandemic and strict safety measures on the treatment of patients with PAD at our tertiary care hospital.

## 2. Materials and Methods

This study employed a retrospective, single-center, observational cohort design from a prospectively maintained database at a large tertiary care hospital in Austria.

Inclusion criteria: we investigated clinical data from all patients older than 18 years admitted to our hospital with vascular disorders between March 16 and 7 December 2020.

Exclusion criteria: patients younger than 18 years or admitted to our hospital with a diagnosis other than vascular disorders.

The first LD in Austria was implemented from March 16 to 4 May 2020. We defined the time from May 5 to 16 November 2020, as the post-LD period. Then, the second LD in Austria was implemented from November 17 to 7 December 2020. We also compared our findings with retrospectively collected clinical data sourced from VPs between March 16 and 7 December 2019. In addition, we collected data from all VPs admitted to our hospital between March 16 to 7 December 2019 and March 16 to 7 December 2020.

All private information of the patients were de-identified. This study was registered with the ClinicalTrials.gov Identifier NCT04838093. Patients’ clinical status was assessed using the functional classification system of Rutherford [7].

The endpoint of the present study was the impact of the COVID-19 pandemic on the treatment of patients with PAD in a tertiary care hospital, which was investigated by determining the number of emergency hospital admissions, acutely performed open surgeries, endovascular interventions and amputations following the implementation of institutional and governmental safety measures.

### 2.1. Cohorts

We compiled the following cohorts:

PAD patient cohort: patients with PAD consecutively admitted to our hospital from March 16 to 7 December 2020.

Control cohort: patients with PAD consecutively admitted to our hospital from March 16 to 7 December 2019.

**Figure 1:**
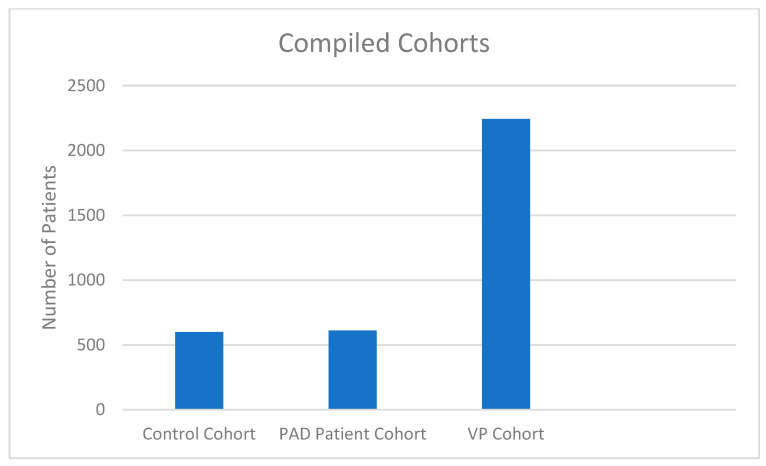
Compiled cohorts. Control cohort: patients with PAD consecutively admitted to our hospital from March 16 to 7 December 2019; PAD patient cohort: patients with PAD consecutively admitted to our hospital from March 16 to 7 December 2020; VP cohort: patients with vascular disorders consecutively admitted to our hospital from March 16 to 7 December 2020.

Vascular patient cohort: patients with vascular disorders consecutively admitted to our hospital from March 16 to 7 December 2020 (Figure 1).

### 2.2. SARS-CoV-2 Testing

Beginning in March 2020, nasal or pharyngeal respiratory swabs were routinely collected from each patient admitted to our hospital and repeated regularly at 48-h intervals during the entirety of their inpatient stay. Testing was performed in the Department of Laboratory Medicine, Medical University of Vienna, Austria, using real-time polymerase chain reaction. The comparability of the results of all test methods used was confirmed by participation in international quality control ring trials [8].

### 2.3. Statistics

Descriptive statistics were used to summarize baseline characteristics and outcome measures. Continuous variables are presented as median, minimum and maximum values due to skewed distributions. Differences between two groups were revealed using Wilcoxon’s rank-sum test. Categorical variables are presented as frequencies and percentages and corresponding 95% confidence intervals (CIs) were calculated according to the method of Wilson [9]. Categorical variables were compared with the chi-squared test or Fisher’s exact test in the case of sparse data. A two-sided significance level of 0.05 was adopted for all statistical tests. Statistical analysis was performed using the statistical software SAS© (SAS Institute Inc., version 9.4, Cary, NC, USA).

## 3. Results

### 3.1. Patient Characteristics

Clinical characteristics and SARS-CoV-2-related characteristics of all patients with vascular disorders that were admitted to our hospital during the observation period are depicted in Table 1.

Within the observation period between March 16 and 7 December 2020, a total of 611 patients with PAD [374 (61%) men, 237 (39%) women] were admitted to our hospital departments. Patient characteristics are depicted in Table 2. All elective admissions occurred one week after one or more outpatient hospital visits.

### 3.2. Impact of COVID-19 Pandemic on the Clinical Treatment of Patients with PAD

Overall admission numbers for patients with PAD were higher in 2020 (n = 611; 27%) than in 2019 (n = 599; 23%) (*p* = 0.7301). The number of admissions during the first LD in 2020 (n = 94; 32%) was significantly lower than that during the same time period in 2019 (n = 136; 26%) (*p* = 0.0056). The number of admissions during the post-LD in 2020 (n = 466; 22%) was somewhat higher than that during the same time period in 2019 (n = 414; 22%) (*p* = 0.0796). This measurement was not significantly different during the second LD 2020 compared to the same time period in 2019 (*p* = 0.8415). Data are depicted in Table 1 and Table 2.

The number of admitted female patients was higher during the entire observation period in 2020 (n = 237; 39%) as compared with 2019 (n = 217; 36%), but the difference was not significant (*p* = 0.3577).

According to our findings, patients admitted to our departments in 2020 [median age: 68 (1–99) years] were significantly younger than those admitted in 2019 [median age: 70 (25–91) years] (*p* = 0.0209).

We observed a highly significant increase in the absolute number and in the proportion of emergency admissions in 2020 (n = 79; 13%) relative to in 2019 (n = 50; 8%) (*p* < 0.0098). There was also a highly significant trend toward greater emergency admissions during the first LD period (n = 24; 25%) when compared to the same time period in 2019 (n = 8; 6%) (*p* < 0.0001). Even during the second LD, we still observed an increased proportion of emergency admissions (n = 4; 8%) relative to during the same time period in 2019 (n = 2; 4%), though the difference was not significant (*p* = 0.4285). The first LD also involved higher proportions of emergency admissions (n = 24; 25%) than the post-LD period (n = 51; 11%) and the second LD (n = 4; 8%) (*p* = 0.0007).

The number of patients recorded with advanced stage PAD differed between 2020 and 2019. Specifically, a proportional increase in cases of stage 4–6 PAD, defined by Rutherford classification [7], was observed during the entire observation period in 2020 (n = 122; 20%) as compared with that in 2019 (n = 50; 8%). During the first LD in 2020 (n = 26; 28%) we observed a similar proportional increase as compared with 2019 (n = 9; 6%) (*p* < 0.0001), which persisted even during the post-LD period (n = 90; 19%) in 2020, compared to (n = 38; 9%) in 2019 (*p* < 0.0001), albeit appearing somewhat less pronounced during the second LD (n = 6; 12%) in 2020 compared to 2019 (n = 3; 5%) (*p* = 0.3243).

In 2020 (n = 586 in 422 patients), we carried out slightly more operations than we did in 2019 (n = 461 in 368 patients). During the first LD, the absolute number of performed operations (n = 75 in 51 patients) was smaller than that in 2019 (n = 107 in 74 patients). However, absolute number of performed operations increased during post-LD (n = 462 in 347 patients), and second LD (n = 49 in 24 patients) periods in 2020 relative to the respective time periods in 2019 (n = 321 in 264 patients and n = 33 in 30 patients). Treatment data of patients with PAD are depicted in Table 2. In patients receiving both endovascular and open surgery every treatment modality was considered separately.

During the pandemic period in 2020, the risk for unsuccessful revascularization in PAD patients with critical limb ischemia almost quadrupled (n = 43; 7%) in comparison with that recorded during 2019 (n = 10; 2%) (*p* < 0.0001). This increase was particularly apparent when comparing the first LD period in 2020 (n = 14; 15%) and the same period in 2019 (n = 3; 2%), with a greater than fourfold increase in the amputation rate recorded (*p* = 0.0003). An increased risk for amputation was also observed for the post-LD (n = 26; 6%) and second LD (n = 3; 6%) periods in 2020, relative to the respective time periods in 2019 (n = 5; 1%; *p* = 0.0004, and n = 2; 4%; *p* = 1). Each amputated patient was considered as one case regardless of number of follow-up resections. Data are depicted in Table 3.

During the COVID-19 pandemic in 2020, the overall mortality rate among patients with PAD decreased to 74 per 1000 patients, as compared with 154 per 1000 patients in the same period in 2019. The 30-day mortality rate was 3.4% (95% CI: 2.2–5.1%) in 2020 and 2.3% (95% CI: 1.3–3.8%) in 2019.

### 3.3. SARS-CoV-2 Prevalence among VPs

To determine the frequency of SARS-CoV-2 infection among VPs, we assessed the prevalence of this viral infection in our patient population. Patient characteristics are depicted in Table 1.

During the observation period, a total of five SARS-CoV-2 infections were detected, with infections in three men and two women. In one asymptomatic patient with extracranial artery disorder, SARS-CoV-2 infection was detected one day before elective admission, which was postponed. In two other cases, an asymptomatic infection was detected two days after elective admission, and both patients only noticed a mild fever. Meanwhile, two symptomatic cases were admitted to an acute setting with confirmed SARS-CoV-2 infection. One patient was found to have an acute myocardial infarction, and one patient was in critical condition with respiratory distress in urgent need of extracorporeal membrane oxygenation. These individuals were co-diagnosed with coronary artery disease and pulmonary comorbidities. No SARS-CoV-2-related deaths were observed, and all five patients achieved viral clearance (polymerase chain reaction cycle threshold > 35) at 12 to 27 days after testing positive.

The proportion of VPs with positive SARS-CoV-2 test results during the first LD period was 0.34% (95% CI: 0.06–1.9%). Meanwhile, the calculated proportion for the same in the post-LD period was 0.17% (95% CI: 0.06–0.5%), and that for the second LD period was 0.50% (95% CI: 0.09–2.75%). The proportion of patients with positive SARS-CoV-2 test results during the entire observation period was 0.22% (95% CI: 0.10–0.52%), but only three patients (out of 2,243) tested positive at admission, for a proportion of 0.13% (95% CI: 0.05–0.39%). No SARS-CoV-2 infection was detected in admitted patients with PAD.

## 4. Discussion

**Figure 2:**
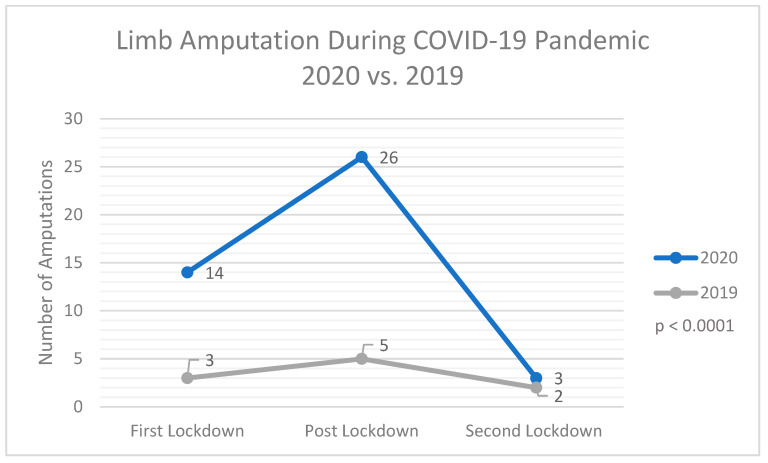
Numbers of limb amputations performed during the first LD, post-LD, and second LD periods in 2020 in comparison with similar time periods in 2019.

As a matter of principle, patients with PAD require continuous medical care to avoid limb-threatening situations. However, recent findings have suggested that the heightened focus on pandemic management has disadvantaged this patient population. Our study assessed patients with PAD in the post-LD period in Austria, defined as May 5 to 16 November 2020. Our data revealed that a significantly higher number of limb amputations were performed on patients with PAD during the COVID-19 pandemic compared to 2019 (*p* < 0.0001) (Figure 2).

This was in line with Schuivens [10] and Sena and Galleli [6], who suggested that patients faced greater difficulty in reaching health care providers because of the exhaustive measures adopted to mitigate the spread of SARS-CoV-2 and patients’ fear of infection. Aside from that, major amputation, one of the most feared complications of PAD, has a tremendous impact on the autonomy and independence of patients and is associated with a high 12-month mortality rate of nearly 50% [11]. Thus, 19 (3%) of the patients who underwent major amputation during the pandemic period of 2020, were expected to die within the next year, which was a significant clinical concern.

Our findings indicated an increased number of overall admissions occurred during the COVID-19 pandemic among the PAD patient population. More precisely, the number of admissions decreased significantly during the first LD period (*p* = 0.0056), consequently increased during the post-LD period (*p* = 0.0796), and then remained consistent during the second LD period. This was in line with data reported by Stabile et al., who recorded a reduced hospitalization rate among patients with chronic limb-threatening ischemia during the first LD period [12]. Moreover, in contrast to Mesnier et al., who reported a reduced hospitalization rate among patients with coronary artery disease in the first four weeks of the post-LD period [13], our data was collected during 16 weeks post-LD and a second LD from patients with PAD.

According to our observations, patients admitted during the COVID-19 pandemic were significantly younger than those admitted in 2019 (*p* = 0.0209). More interestingly, patients admitted post-LD were younger than those admitted during the first LD. This was in line with the data presented by Mesnier et al., in which the proportion of elderly patients decreased in the post-LD period [13]. We considered this finding to be an aftereffect of the strict LD, which interrupted routine follow-ups, resulting in disadvantaged younger patients.

There has recently been growing evidence that pre-existing cardiovascular disease is associated with worse clinical outcomes in the case of SARS-CoV-2 infection [5]. Further data have suggested a possible relationship between SARS-CoV-2 and endothelial damage, leading to increased thrombotic events [14,15]. Although cardiovascular research is currently investigating the impacts of SARS-CoV-2, to our knowledge, there is no consistent evidence suggesting that patients with PAD have a greater susceptibility to SARS-CoV-2 infection. According to our study, no PAD patients were infected with SARS-CoV-2 during the observation period. Therefore, we recommend patient care be continued while emphasizing the use of appropriate personal protective equipment, as indicated by Eilenberg et al. [16]. From our point of view, this is the most effective strategy to reduce transmission within the health care setting until there has been thorough vaccination of both patients and health care personnel. Moreover, we must consider that the low frequency of SARS-CoV-2 infection might be because this patient population complies with safety precautions and makes greater efforts to protect themselves.

In addition, there was a significant increase in emergency admissions during the pandemic and an increased proportion of stage 4–6 PAD, according to Rutherford classification [7], especially in younger individuals. Additionally, while some have suggested that increased SARS-CoV-2-related mortality rates exist among patients with cardiovascular diseases, we did not observe any such condition-specific rise in SARS-CoV-2-related mortality [17]. Instead, in line with Bozzani et al. [18], Viswanathan et al. [19] and Lou et al. [20], we observed increased PAD-related complications, including limb amputation and perioperative mortality.

This raises concerns about the disproportionate prioritization of COVID-19-related conditions leading to insufficient health care services provided to others. We are aware of the necessity of smart safety measures to prevent uncontrolled viral spread, but we also believe that the definition of standards to simplify patient access to health care facilities, even in times of global pandemics, would prevent the unfavorable outcomes observed during and after the first pandemic-related LD in 2020. In addition, as we have demonstrated, during the 16 weeks of the post-LD period, the real impact of extensive LD presented itself in the post-LD period with a backlog of acute-on-chronic conditions requiring treatment. This trend appeared to persist even long after the peak of the pandemic had passed. As reflected by Di Giovanni et al. [21] and Lopez-de-Andres et al. [22], patients with a high number of comorbidities, including PAD, are at higher risk for amputation. Therefore, in the case of further pandemic outbreaks, to avoid undertreatment of PAD, this patient population should be continuously monitored, because the patients suffer more from the progression of PAD than from expected pandemic-related conditions. We believe that the data provided by our study could inform decision-makers in the creation of public safety policies for critical infrastructure, such as hospitals.

Limitations: One limitation of our study was that we could not specify the cause of every amputation in detail, for example, regarding the course of treatment. Another limitation was that there could be a high number of unrecorded at-home infections with SARS-CoV-2 that we could not include in this study. Further investigations using nationwide registry data would be necessary to address this matter more precisely.

Conclusion: This study highlights the fact that the pandemic-related obstacles that must be faced not only include hypercoagulation- and infection-related deaths, but also the neglect of noninfected patients dealing with chronic conditions in need of continuous health care, especially patients with PAD.

## Figures and Tables

**Table 1:** Clinical and SARS-CoV-2 related characteristics of patients with vascular disorders.

	Whole Observation Period		March–May		May–November		November–December	
			First LD		Post-LD		Second LD	
Year	2020	2019	2020	2019	2020	2019	2020	2019
Total No. of admitted vascular patients	2243	2637	290	529	1751	1903	202	205
No. of patients with PAD	611 (27%)	599 (23%)	94 (32%)	136 (26%)	466 (27%)	414 (22%)	51 (25%)	49 (24%)
Sex								
Male	1336 (60%)	1529 (58%)	178 (61%)	316 (60%)	1030 (59%)	1089 (57%)	128 (63%)	127 (62%)
Female	907 (40%)	1107 (42%)	112 (39%)	213 (40%)	721 (41%)	814 (43%)	74 (37%)	78 (38%)
Median age, years (Range)	70 (18–100)	71 (18–99)	71 (22–100)	71 (18–99)	70 (18–97)	70 (18–99)	70 (29–94)	71 (18–92)
COVID-19 related characteristics								
No. of tests performed	13,156		1697		10,261		1184	
Positive SARS-CoV-2 test	5 (0.2%)		1 (0.3%)		3 (0.2%)		1 (0.5%)	
Sex								
Male	3 (60%)		0		2		1	
Female	2 (40%)		1		1		0	
Diagnosis ^1^			Extracran. artery disorder		Cardiac disorder		Cardiac disorder	

Categorical variables are presented as frequencies and percentages. Continuous variables are presented as medians with minimum and maximum values. LD: lockdown, PAD: peripheral artery disease. ^1^ Admission diagnosis of patients positive for SARS-CoV-2 infection.

**Table 2:** Characteristics of patients with peripheral artery disease.

	Whole Observation Period		March–May		May–November		November–December	
			First LD		Post-LD		Second LD	
Year	2020	2019	2020	2019	2020	2019	2020	2019
No. of admitted Patients with PAD	611 (100%)	599 (100%)	94 (100%)	136 (100%)	466 (100%)	414 (100%)	51 (100%)	49 (100%)
2019 vs. 2020	*p* = 0.7301		*p* = 0.0056		*p* = 0.0796		*p* = 0.8415	
Sex								
Male	374 (61%)	382 (64%)	63 (67%)	84 (62%)	279 (60%)	262 (63%)	32 (63%)	36 (73%)
Female	237 (39%)	217 (36%)	31 (33%)	52 (38%)	187 (40%)	152 (37%)	19 (37%)	13 (27%)
Median age, years (Range)	68 (18–99)	70 (25-91)	75 (57–93)	70 (30–93)	72 (18–88)	70 (25–95)	70 (29–94)	72 (46–91)
Inpatient admission								
Elective	532 (87%)	549 (92%)	70 (75%)	128 (94%)	415 (89%)	374 (90%)	47 (92%)	47 (96%)
Emergency	79 (13%)	50 (8%)	24 (25%)	8 (6%)	51 (11%)	40 (10%)	4 (8%)	2 (4%)
2019 vs. 2020	*p* = 0.0098		*p* < 0.0001		*p* = 0.5329		*p* = 0.4285	
Median Hospital stay, days (Range)	6 (1–117)	7 (1–195)	6 (1–57)	6 (1–195)	6 (1–117)	6 (1–179)	5 (1–25)	7 (1–108)
Rutherford classification								
1–3	487 (80%)	549 (92%)	68 (72%)	140 (94%)	374 (81%)	404 (91%)	45 (88%)	52 (95%)
4–6	122 (20%)	50 (8%)	26 (28%)	9 (6%)	90 (19%)	38 (9%)	6 (12%)	3 (5%)
2020 vs. 2019	*p* < 0.0001		*p* < 0.0001		*p* < 0.0001		*p* = 0.3243	
Comorbidities								
Hypertension	496 (81%)	464 (77%)	77 (82%)	106 (78%)	380 (81%)	322 (78%)	39 (76%)	36 (73%)
Diabetes	215 (35%)	203 (34%)	26 (28%)	63 (46%)	170 (36%)	128 (31%)	19 (37%)	12 (24%)
Dyslipidemia	365 (60%)	336 (56%)	47 (50%)	90 (66%)	283 (61%)	221 (53%)	35 (69%)	25 (51%)
Renal failure	38 (6%)	36 (6%)	7 (7%)	13 (9%)	28 (6%)	21 (5%)	3 (6%)	2 (4%)
No. of admitted Patients with PAD	611 (100%)	599 (100%)	94 (100%)	136 (100%)	466 (100%)	414 (100%)	51 (100%)	49 (100%)
Treatment ^1^								
Endovascular	95 (15%)	126 (21%)	13 (14%)	41 (30%)	69 (15%)	82 (20%)	13 (25%)	3 (6%)
Open repair	491 (80%)	335 (56%)	62 (66%)	66 (48%)	393 (84%)	239 (58%)	36 (71%)	30 (61%)
Conservative	189 (31%)	231 (39%)	43 (46%)	62 (46%)	119 (25%)	150 (36%)	27 (53%)	19 (39%)

Categorical variables are presented as frequencies and percentages. Continuous variables are presented as medians with minimum and maximum values. LD: lockdown, PAD: peripheral artery disease. ^1^ Absolute numbers and proportions of performed interventions are presented. The proportions given refer to absolute numbers of patients with PAD. In patients receiving both endovascular and open surgery every treatment modality is presented separately. Each patient treated conservatively is presented as one case.

**Table 3:** Number of limb amputations and survival in patients with peripheral artery disease.

	Whole Observation Period		March–May		May–November		November–December	
			First LD		Post-LD		Second LD	
Year	2020	2019	2020	2019	2020	2019	2020	2019
Amputations	43 (7%)	10 (2%)	14 (15%)	3 (2%)	26 (6%)	5 (1%)	3 (6%)	2 (4%)
2019 vs. 2020	*p* < 0.0001		*p* = 0.0003		*p* = 0.0004		*p* = 1	
Minor	24 (4%)	2 (<1%)	7 (7%)	0	15 (3%)	2 (<1%)	2 (4%)	0
Major	19 (3%)	8 (1%)	7 (7%)	3 (2%)	11 (2%)	3 (<1%)	1 (2%)	2 (4%)
Survival								
Overall mortality	45 (9%)	92 (15%)	15 (16%)	28 (21%)	27 (6%)	58 (14%)	1 (2%)	6 (12%)
30-day mortality	21 (3%)	14 (2%)	8 (8%)	4 (3%)	12 (3%)	9 (2%)	1 (2%)	1 (2%)

Categorical variables are presented as frequencies and percentages. LD: lockdown.

## Data Availability

All data supporting reported results are available upon request.
